# Genetic variability of arrhenotokous and thelytokous *Venturiacanescens* (Hymenoptera)

**DOI:** 10.1007/s10709-012-9657-6

**Published:** 2012-05-22

**Authors:** Irene Mateo Leach, Steven Ferber, Louis van de Zande, Leo W. Beukeboom

**Affiliations:** 1 Evolutionary Genetics, Centre for Ecological and Evolutionary Studies, University of Groningen, PO Box 11103, 9700 CC Groningen, The Netherlands; 2 Present Address: Experimental Cardiology, University Medical Center Groningen, Ant. Deusinglaan 1, 9713 AV Groningen, The Netherlands

**Keywords:** *Venturia canescens*, Genetic variability, Linkage, Microsatellites, Recombination, Reproductive mode

## Abstract

The ichneumonid wasp *Venturia canescens* (Hymenoptera) has been studied extensively for foraging behaviour and population dynamics of sexually (arrhenotokous) and parthenogenetically (thelytokous) reproducing individuals. Here we report the development of a set of microsatellite markers for *V.canescens* and use them to show that arrhenotokous individuals have more genetic variability than thelytokous ones, which are even homozygous for all tested loci. Crosses between arrhenotokous individuals suggested one marker, *Vcan071*, to be linked with the Complementary Sex Determiner (CSD) locus and one, *Vcan109,* with the Virus Like Protein (*vlp*-*p40*) locus. The genome size of *V. canescens* was estimated to be 274–279 Mb. We discuss how both reproductive modes can give rise to the observed genetic variability and how the new markers can be used for future genetic studies of *V. canescens*.

**Electronic supplementary material:**

The online version of this article (doi:10.1007/s10709-012-9657-6) contains supplementary material, which is available to authorized users.

## Introduction

Genomic information and genetic tools are essential for the genetical analysis of biological traits. They are available for many plant and animal species that are of economical value such as crops and life stock. However, very little genetic and genomic information is available for parasitoid wasps. Genetic marker linkage maps have been published for few parasitoids (Antolin et al. 1996; Laurent et al. 1998; Gadau et al. 1999, 2008; Holloway et al. 2000; Beukeboom et al. 2010; Niehuis et al. 2010). Molecular markers are also essential for annotating full genome sequences, such has recently been done for the wasp *Nasonia* (Werren et al. 2010). Marker development and genomic information provides the first step towards the genetic analysis of parasitoid behaviour and life history traits. This is needed to gain a better understanding of the behavioural ecology and evolution of parasitoids and their use in biological control (Roderick and Navajas 2003; Wajnberg et al. 2008).

The ichneumonid wasp *Venturia canescens* (Hymenoptera) has been studied extensively in behavioural ecology, such as for testing optimal foraging and energy allocation (e.g. Amat et al. 2006; Thiel et al. 2006; Lucchetta et al. 2008; Metzger et al. 2008; Liu et al. 2009; Desouhant et al. 2010; Lukas et al. 2010; Pelosse et al. 2010). *V. canescens* is a parasitoid of lepidopteran larvae that occur on figs and almonds in southern Europe (Driessen and Bernstein 1999). As all Hymenoptera, it has haplodiploid reproduction. Sexual reproduction under haplodiploidy is called arrhenotoky: unfertilized eggs develop into haploid males and fertilized eggs into diploid females. Asexual reproduction is called thelytoky: diploid females develop parthenogenetically from unfertilized eggs and males are absent. *V. canescens* is a rare example of a species that has both sexually and parthenogenetically reproducing individuals that occur in sympatry (Schneider et al. 2002). Arrhenotokous females have meiotic oogenesis that allows for recombination. In contrast, thelytokous females have a form of automictic parthenogenesis that allows for some variation among offspring, but will lead to an irreversible increase of homozygosity in populations over time (for details see Beukeboom and Pijnacker 2000; Mateo Leach et al. 2009b).

Schneider et al. (2002) studied the geographical distribution and genetic diversity of arrhenotokous and thelytokous populations of *V. canescens* in the Côte d’Azur (France) using both nuclear and mitochondrial markers. Analysis of the genetic structure of the populations revealed one widespread thelytokous lineage and a few rare ones with high genetic similarity to arrhenotokous individuals. In a later study, Schneider et al. (2003) found similar results with wasps collected from a 500 km transect along the Rhône Valley in Southern France. At that time only Amplified Fragment Length Polymorphism (AFLP) nuclear markers and Restriction Fragment Length Polymorphism (RFLP) mitochondrial markers were available which restricted the analysis of population structure. This lack of suitable markers for population genetic analysis prompted us to develop a series of microsatellite markers that are more efficient and reliable for intraspecific studies.

Another field in which application of molecular markers is particularly rewarding is that of locating genes underlying specific traits, such as the sex determining loci in Hymenoptera. Many hymenopteran species have a form of complementary sex determination in which sex depends on the allelic composition of a single or multiple loci (Cook 1993; van Wilgenburg et al. 2006; Heimpel and de Boer 2008). The CSD locus has been mapped in a number of hymenopteran species (Antolin et al. 1996; Holloway et al. 2000; Gadau et al. 2001) and cloned from the honey bee (Beye et al. 2003). However, the phylogenetic distribution of single and multiple loci CSD within the Hymenoptera is still far from clear, and the *csd* gene has not yet been isolated from any other species than the honey bee. *V. canescens* has been shown with genetic crosses to have single locus CSD (Beukeboom 2001). Another commonly used locus in *V. canescens* for genetic studies is the Virus Like Particle locus (*vlp*-*p40*). VLPs are important in parasitoid-host interactions as they coat the parasitoid’s egg preventing it to be detected and destroyed by the immune system of the host (Fedderson et al. 1986; Hellers et al. 1996; Reineke et al. 2006). *vlp*-*40* Is a gene with two allelic forms differing in the presence of a short tandem repeat of 54 bp in the coding region (*vlp*+ and *vlp*−; Hellers et al. 1996). These two variants have been used to characterize arrhenotokous and thelytokous strains form the field and in the lab (Beck et al. 2001; Schneider et al. 2003). In this study we determine its linkage to the newly developed microsatellite markers.

Studies of biological traits in *V. canescens*, such as life history and foraging behaviour, can be taken further if genomic information is available, as that allows for uncovering the genetic basis of these traits. The aim of our study is to provide some basic genomic information of *V. canescens*, including its genome size and a set of microsatellite markers that can be useful for future biological studies of this species. This set of markers complements a previously developed set of 59 microsatellites for this species (Butcher et al. 2000), which proved to be uninformative for our studies. We apply these markers to investigate in more detail the genomic variability of arrhenotokous and thelytokous wasps using individuals from the same populations as Schneider et al. (2002). In addition, we use sequence data of the mitochondrial *COI* gene to support the observed differences in genetic variability between the two reproductive modes.

## Materials and methods

### Physical genome size estimation

The diploid physical genome size of *V. canescens* was determined by standard flow cytometry. Adult female wasp heads were homogenized in Galbraith buffer (21 mM MgCl_2_, 30 mM tri-Na citrate dihydrate, 20 mM MOPS, 0.1 % Triton X-100, 1 mg/l RNase, pH 7.2), filtered (50 μm), stained overnight in propidium iodide (Sigma, St. Louis, Missouri, USA) and loaded on a LFRII flow cytometer (BD BioSciences, Franklin Lakes, NJ, USA). As size standard we used whole body cells of *Drosophila melanogaster* and the hymenopteran *Nasonia vitripennis* whose haploid genome sizes are known to be 176 and 312 Mb respectively (Wilfert et al. 2007; Werren et al. 2010).

### Microsatellite marker development

Genomic DNA from 10 arrhenotokous female wasps collected at Mont Boron in 1999 (Schneider et al. 2002) was extracted using a standard proteinase K/salt-chloroform Protocol (Sambrook et al. 1989). Presence and quality of DNA was checked on a 1 % agarose gel. A microsatellite genomic library was constructed according to the SSR capture enrichment method of Connel et al. (1998) by Baseclear B.V. (Leiden, the Netherlands). DNA was fractionated by nebulization and ligated to adapters (oligo AP-11: 5′-CTCTTGCTTAGATCTGGACTA-3′ and oligo AP-12: 5′-TAGTCCAGATCTAAGCAAGAGCACA-3′). Selection for a subset of adapter ligated fragments was done by PCR amplification with AP-11 adaptor as primer. Biotinylated repeat oligos (GAG, CTA, CAT, CAC, GAG and CAAC) were used to hybridize and isolate repeat sequences from the fragmented DNA. The fragments containing repeat sequences were isolated using streptavidin-coated paramagnetic beads (Promega). One microliter of the selected fragments was amplified using AP-11 oligo, ligated into pCRII vectors (Invitrogen) and transformed into *Escherichia coli* DH10B cells according to Sambrook et al. (1989). Positive clones were selected for PCR amplification using M13 forward or reverse primers and the resulting fragments were sequenced on an Applied Biosystems DNA analyzer 3730 using Big Dye terminator V3.1 (Applied Biosystems). To characterize the repeats in the clone sequences, these were further analyzed using the Tandem Repeat Finder software (Benson 1999). On the selected sequences, primers were designed with software PRIMER 3 (Rozen and Skaletsky 2000).

### Linkage analysis of the *csd* and *vlp-p40* loci

For the linkage analysis of the *csd* locus a brother-sister cross was used similar to Beukeboom (2001) to create linkage disequilibrium between the *csd* locus and linked microsatellites. A microsatellite locus linked to the *csd* locus will be heterozygous in diploid females and homozygous in diploid males that carry two sex alleles that are identical by descent. All offspring were genotyped for 15 polymorphic microsatellites and male ploidy was determined with flow cytometry. For the linkage analysis of the *vlp*-*p40* locus we crossed two strains that were known to harbour different *vlp*-*p40* alleles. Segregation of the *vlp*-*p40* and microsatellite alleles was scored in haploid sons based on the known linkage phase in their heterozygous mothers. For amplification of the *vlp*-*p40* locus we used specific primers (VLPF 5′-CTCAATATGTGGGGTGGTGG-3′ and VLPR 5′-TCGCAGTGGCTTGTCAGAGT-3′) (Hellers et al. 1996). PCR reactions for *vlp*-*p40* were performed in 0.4× PCR buffer magnesium free (Promega) supplemented to a final concentration of 1 mM MgCl2, 0.08 mM dNTPs (Roche), 0.2 pmol/ml of each primer, 0.4 units of Taq polymerase (Promega) and approximately 5 ng of template DNA. The PCR profile was 1 cycle of 2 min at 94 °C followed by 35 cycles of 1 min at 94 °C, 1 min at 55 °C, 1 min at 72 °C and a final cycle of 10 min at 72 °C. PCR products were checked on an 1.5 % agarose gel. Linkage was tested with a Chi-square test with Yates correction and a significance level of *p* < 0.01.

### Microsatellite variation

**Fig. 1:**
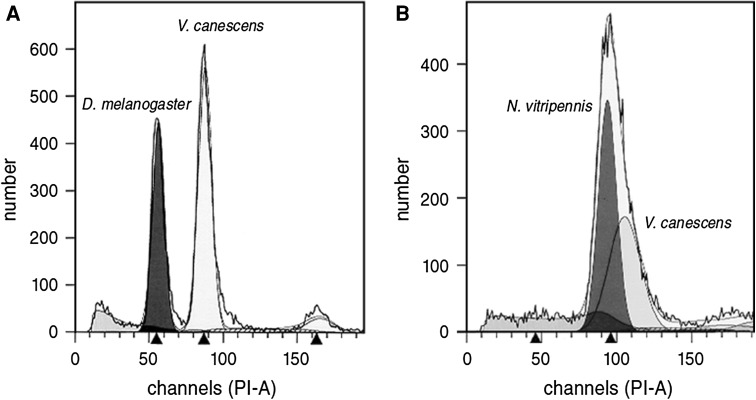
Flow cytometric analysis of the *V. canescens* genome. The known genome sizes of (**a**) *D. melanogaster* and (**b**) *N. vitripennis* (*dark grey shading*) are used for comparison. Cell number is indicated on the y-axis and propidium-iodide (*PI-A*) concentration as a measure of DNA concentration on the x-axis. Genome size is estimated to be 274–279 Mb

Genomic DNA was extracted from the abdomen of 22 arrhenotokous and 7 thelytokous females from field collected populations (SOM Table 5). One female per population was used because wasps collected from a single site had a chance of being genetically related (see Schneider et al. 2002 for details). Fifteen microsatellite markers were selected based on reliability in amplification and variability for genotyping these 29 individuals; 26 wasps from the same populations as the ones used in Schneider et al. (2002), two additional populations from France, and one from Spain (SOM Figure 1; SOM Table 1).

The genetic diversity of microsatellite markers was quantified per reproductive mode using number of alleles, heterozygosity, allelic richness and allele frequencies observed in each group using Fstat (Goudet 2001). To determine whether there was a significant difference between the genetic diversity of the two reproductive modes, we performed a Wilcoxon matched pairs test on a set of 15 microsatellites to compare the observed heterozygosities and a *t* test to compare the allelic richness between both reproductive modes using Statistica (StatSoft, OK, USA). An unrooted UPGMA tree using Nei’s standard genetic distance *DS* (Nei 1987) was constructed with the software package POPULATIONS (available at: http://bioinformatics.org/~tryphon/populations/). The genetic variability per individual was calculated as the number of heterozygous markers divided by the number of markers amplified. The mean heterozygosity per reproductive mode was compared with a two sample *t* test using the statistical package Statistix 4.0 analytical software.

### Mitochondrial DNA analysis

A 449-bp fragment of the mitochondrial Cytochrome Oxidase I (*COI*) gene was amplified by PCR with primers *COI Vcan* F 5′-GGTTTGGCTCTATTGGGATAA-3′ and *COI Vcan* R 5′-AAAATGTTGAGGGAAAAATGTTAGA-3′. PCR reactions were carried out in a 25 μl reaction volume containing approximately 5 ng of DNA, 1× PCR buffer magnesium free (Promega), 2.5 mM MgCl2, 0.2 mM dNTPs (Roche), 0.2 μM of each primer and 0.4 units of Taq polymerase (Promega). The cycling conditions were 1 min denaturation at 94 °C, followed by 35 cycles of 1 min denaturation at 94 °C, 1 min annealing at 55 °C, and 1 min and 30 s extension at 72 °C, ending with a final extension at 72 °C for 5 min. PCR products were purified with isopropanol and sequenced in one direction using primer *COI Vcan* F with fluorescent Big Dye terminator (Applied Biosystems, Warrington, UK) on a 377 DNA sequencer from Applied Biosystems. Mitochondrial *COI* sequences were aligned using ClustalX (Thompson et al. 1997) and haplotype diversity was calculated using DnaSP version 4.10 (Rozas et al. 2003). Genetic distances were calculated with DNADIST using the software package PHYLIP version 3.6 (Felsenstein 2004) to construct a distance tree.

## Results

### Genome size

The DNA content of female *V. canescens* head cells was 1.56 compared to *D. melanogaster* cells and 0.89 compared to *N. vitripennis* (Fig. 1). The physical size of the genome of *V. canescens* was therefore estimated to be between 274 Mb (176 Mb × 1.56) and 279 Mb (312 Mb × 0.89).

**Fig. 2:**
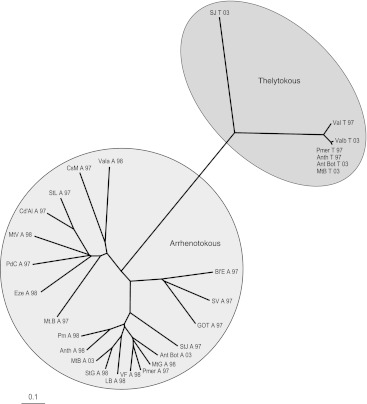
Distance tree illustrating the relationships among arrhenotokous and thelytokous individuals of *V. canescens*. The tree was based on Nei’s standard genetic distance *DS* (Nei 1987) using 15 microsatellite loci. *Colours* indicate the haplotype of each individual. Location abbreviations are listed in SOM Table 1

### Microsatellite marker development

Of 287 sequenced fragments from the enriched library, 143 sequences (49.8 %) contained a repeat motif of di-, tri- or tetranucleotides. 50 % Of the repeat motif containing sequences had one microsatellite motif, others had two (25 %), three (17 %) or four and more (8 %) repeat types. 53 % Of the microsatellites were dinucleotides, 32 % trinucleotides, and 15 % tetranucleotides. The proportion of perfect repeats (according to Weber’s definition, Weber 1990) was 46.6 %. Among the dinucleotide motifs the most common were TC/AG (39 %) and CT/GA (24.8 %) (SOM Table 2). Many different trinucleotide repeats were represented in the library: CAA/GTT (9 %) and AGA/TCT (7 %) being the most abundant ones. We also found a large diversity of tetranucleotide repeats of which GTGC/CACG and ATCT/TAGA were the most abundant ones (13 %). Our results are in contrast with the most predominant motif found by Butcher et al. (2000), which was GT, but consistent with motifs in other Hymenoptera (Estoup et al. 1993; Pannebakker et al. 2010). From the data summarized in Pannebakker et al. (2010), *Apis mellifera* is the most comparable species to *V. canescens* according to the proportion of microsatellites and type of repeats, followed by the three sister species of *Nasonia*. A total of 56 reliably amplifiable microsatellite markers could be developed (SOM Table 2). These are labelled Vcan060-Vcan115 following the notation of Butcher et al. (2000) and expand their set of 59 microsatellite markers published in Genbank.

### Linkage analysis

We looked for linkage between some of our microsatellite markers and two loci of interest known from *V. canescens*. The *csd* locus was investigated in an inbreeding cross and found to be closely linked with marker *Vcan071* (recombination frequency 6 %, *p* = 0.04, *N* = 31; SOM Table 3). The inheritance of the *vlp*-*p40* locus and 27 microsatellite markers was investigated in haploid offspring of heterozygous females. Only microsatellite marker *Vcan109* showed significant linkage with the *vlp*-*p40* locus after Bonferroni correction with a recombinational distance of 4.7 centiMorgan (χ^2^ = 48, 246, *df* = 1, *p* < 0.0001, *N* = 64) (SOM Table 4). We were not able to construct a complete linkage map of our markers, because sample sizes per family were too small and the number of polymorphic markers that were shared between multiple families too low.

### Microsatellite marker variation

Of the 56 newly developed microsatellites, only 15 were polymorphic in both arrhenotokous and thelytokous individuals. Twenty-two arrhenotokous and seven thelytokous individuals representing 29 populations were screened with these 15 polymorphic microsatellites. Table 1 summarizes the number of alleles, number of individuals amplified, observed heterozygosity and allelic richness per marker and reproductive mode. Allele frequencies are given in SOM Table 6. The number of alleles per locus varied from 2 to 11 in the arrhenotokous individuals and 1–2 in the thelytokous individuals. The arrhenotokous individuals had significantly higher observed heterozygosities than the thelytokous females (*H*_O-arrh_ = 0.48, *H*_O-thel_ = 0, Wilcoxon matched pairs test, *Z* = 3.38, *p* = 0.0007, *N* = 15 microsatellite loci) as well as higher allelic richness values (*A*_arrh_ = 3.73, *A*_thel_ = 1.60, *t* test, *t* = 6.98, *df* = 14, *p* < 0.0001, *N* = 15). Although the number of females tested for each reproductive mode was different, this does not affect the average heterozygosity, but only the variance, as the likelihood of representation of low frequency alleles is smaller in the thelytokous sample. The mean proportion of heterozygous markers per individual (Table 2) is 0.50 (SE = 0.12, *N* = 21) in the arrhenotokous and 0.0 in the thelytokous (SE = 0.0, *N* = 7) sample. The difference in the heterozygosity level is significant (*t* test, *t* = 10.57, *df* = 26, *p* < 0.0001), even though three times as many arrhenotokous individuals were tested, and reveals that thelytokous individuals are completely homozygous for all tested loci. The total number of alleles observed in both modes is 76 alleles in the arrhenotokous group and 24 in the thelytokous one. Thus, proportionally the same number of alleles is present in both reproductive modes, but thelytokous individuals carry more often two similar alleles. There are four alleles that are unique for the thelytokous group.

**Table 1:** Overview of variation at 15 microsatellites for arrhenotokous and thelytokous *V. canescens*

Locus	Allele size	Arrhenotokous				Thelytokous			
		*N*_A_	*N*_ind_	*H*_O_	*A*	*N*_A_	*N*_ind_	*H*_O_	*A*
Vcan061	178–200	7	21	0.67	4.206	2	7	0.00	1.989
Vcan062	250–266	4	21	0.67	3.662	2	7	0.00	1.989
Vcan063	174–186	4	19	0.53	3.030	1	7	0.00	1
Vcan064	277–295	6	21	0.48	3.468	2	6	0.00	2
Vcan065	200–236	11	21	0.86	7.251	2	7	0.00	1.989
Vcan066	240–260	5	21	0.67	3.635	2	7	0.00	1.989
Vcan067	139–160	6	16	0.44	5.334	2	7	0.00	1.989
Vcan069	217–229	5	18	0.50	3.344	1	6	0.00	1
Vcan070	213–230	5	19	0.37	3.388	2	6	0.00	2
Vcan071	228–246	7	12	0.25	5.627	2	6	0.00	2
Vcan097	140–152	3	20	0.40	2.500	2	7	0.00	1.989
Vcan109	189–193	3	19	0.63	2.867	1	7	0.00	1
Vcan110	172–174	2	17	0.00	1.842	1	6	0.00	1
Vcan112	143–161	4	21	0.33	2.731	1	6	0.00	1
Vcan114	237–251	4	19	0.47	3.007	1	6	0.00	1
Mean over loci		5.07		0.48	3.73	1.60		0.00	1.60

Given are allele size range, number of alleles (*N*_A_), number of females tested (*N*_ind_), observed heterozygosity (*H*_O_) and allelic richness (*A*) per locus and reproductive mode

**Table 2:** Genetic diversity per individual

Arrhenotokous				Thelytokous			
Sample	*M*	*H*	*H*/*M*	Sample	*M*	*H*	*H*/*M*
Bl’E A 97	10	6	0.60	Anth T 97	14	0	0
StJ A 97	13	6	0.46	Pmer T 97	14	0	0
Cd’Al A 97	13	9	0.69	Val T 97	13	0	0
StL A 97	14	6	0.43	Ant T 03	15	0	0
CsM A 97	13	5	0.38	Valb T 03	14	0	0
SV A 97	13	6	0.46	MtB T 03	15	0	0
GOT A 97	13	7	0.54	SJ T 03	13	0	0
Mt.B A 97	13	8	0.62				
PdC A 97	14	7	0.50				
Pmer A 97	13	9	0.69				
Eze A 98	15	9	0.60				
LB A 98	14	7	0.50				
MtG A 98	13	5	0.38				
MtV A 98	14	10	0.71				
Anth A 98	15	4	0.27				
Pm A 98	14	8	0.57				
StG A 98	13	4	0.31				
Vala A 98	14	7	0.50				
VF A 98	14	7	0.50				
VV A 98	–	–	–				
Ant A 03	15	5	0.33				
MtB A 03	14	7	0.50				

The number of markers amplified for each individual (*M*), the number of heterozygous markers (*H*) and the heterozygosity per individual as (*H*)/(*M*) are indicated

### Mitochondrial DNA variation

**Fig. 3:**
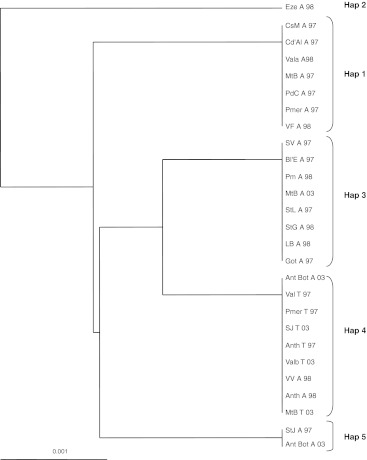
Unrooted cladogram based on the 449 bp mitochondrial *COI* sequence. Genetic distances were calculated using the F84 method in PHYLIP version 3.6 (Felsenstein 2004). *Colours* indicate the haplotypes. Location abbreviations are listed in SOM Table 1

Six single base pair polymorphisms were found in the *COI* gene among 27 samples (20 arrhenotokous and 7 thelytokous) (Table 3) yielding five haplotypes. Haplotypes 1 and 2 differ in four base pairs, but haplotypes 3, 4 and 5 differed from one another in one base pair only. A distance tree of 27 samples is shown in Fig. 3. Haplotypes 1, 2, 3 and 5 were exclusive to arrhenotokous individuals whereas haplotype 4 was the only haplotype present among the thelytokous wasps and occurred in two arrhenotokous individuals. These results expand those of Schneider et al. (2002) who could only distinguish two haplotypes based on RFLPs. The genetic distance analysis (Fig. 2) indicated a clear differentiation between both reproductive modes but there was no obvious clustering of arrhenotokous individuals according to their mitochondrial haplotype or geographical origin.

**Table 3:** Mutation positions of mitochondrial haplotypes

Position						
Haplotype	018	019	020	301	397	419
Hap 1	T	–	C	–	A	A
Hap 2	A	–	–	G	G	A
Hap 3	T	A	T	–	A	A
Hap 4	T	A	T	–	A	G
Hap 5	C	A	T	–	A	A

## Discussion

The physical genome size of the parasitoid wasp *V. canescens* was estimated to be between 274 and 279 Mb which is slightly smaller than the genome of the parasitoid *N. vitripennis* (312 Mb, Werren et al. 2010), and comparable to the hymenopterans *Trichogramma* (246 Mb) and the honey bee (262 Mb) (The Honey bee Genome Consortium 2006; Wilfert et al. 2007).

We developed a set of microsatellite markers for *V. canescens* and used it to compare genetic variability of arrhenotokous and thelytokous individuals. They complement previously reported microsatellite markers (Butcher et al. 2000) which were largely monomorphic in our study populations. A subset of our new markers was polymorphic and we focused on those 15 that were variable in both reproductive modes.

Variation at microsatellite markers in thelytokous wasps is governed by the cytological mode of parthenogenesis (Stouthamer and Kazmer 1994). Beukeboom and Pijnacker (2000) reported central fusion automictic parthenogenesis as the mode of diploidy restoration in thelytokous eggs of *V. canescens*. This mechanism allows recombination to occur in early stages of oogenesis, but leads to an increase of homozygosity. Distal loci will become homozygous over generations while loci near a centromere will remain heterozygous because recombination results only in homozygosity of those loci that are on the exchanged chromatids and cross-overs are reduced near the centromeres. As a consequence, thelytokous individuals are expected to be more homozygous than arrhenotokous ones (Speicher et al. 1965; Crozier 1971; Beukeboom and Pijnacker 2000). The degree of heterozygosity and the allelic richness of 15 microsatellite markers was found to be higher in arrhenotokous than thelytokous individuals, which is in agreement with this prediction. Even more, all thelytokous individuals were completely homozygous which suggest that all markers were on distal portions of the chromosomes and/or that the fixed heterozygous regions near centromeres were small. This result also indicates that recombination is not reduced under thelytokous reproduction in this species, which is in agreement with a study on the thelytokous parasitoid *Lysiphlebus fabarum*, even though not all loci were homozygous in this species (Sandrock and Vorburger 2011). The first study of genetic variability in combination with central fusion was in the cape honey bee, *Apis mellifera capensis*. In this species thelytokous queens did not have reduced recombination (Oldroyd et al. 2008), but thelytokously reproducing workers retained high levels of heterozygosity suggesting strong recombination suppression (Baudry et al. 2004; Oldroyd et al. 2011). Recently, an even more extreme case of reduced recombination in thelytokous individuals was described for the little fire ant *Wasmannia auropunctata* (Rey et al. 2011). Thelytokous queens of this species produce workers via automictic parthenogenesis with central fusion, which apparently do not undergo any homozygosation. The authors suggest that maintenance of genetic variation by heterozygosity as a result of extreme low recombination rates and automixis, confers a selective advantage to asexual reproducing *W. auropunctata*. More studies of thelytokous species are needed to understand how and why recombination is suppressed in some species (cape honey bee, little fire ant) but not in others (parasitoids *V. canescens*, *L. fabarum*).

As shown in the distance tree of Fig. 2, the arrhenotokous and thelytokous individuals are completely separated according to the nuclear marker composition. There are four unique alleles in the thelytokous populations that are not found among the arrhenotokous individuals. There are five mitochondrial haplotypes that showed an accurate association with reproductive mode: thelytokous individuals had exclusively haplotype 4, which was shared with arrhenotokous individuals among which also other haplotypes were present. The most likely explanation for this pattern among our samples is a single origin of thelytoky from arrhenotokous individuals with haplotype 4. Subsequent homozygosation of the genome has likely resulted in the rapid nuclear differentiation of the thelytokous individuals.

We looked for possible linkage of the microsatellite markers with two known loci of *V. canescens*. The sex determination locus *csd* appears to be associated with marker *Vcan071*, as the number of homozygous diploid males for this microsatellite locus is significantly deviant from expected values (χ2 (GOF) = 5.88; *df* = 1; 0.01 < *p* < 0.025) corresponding to a recombination distance of 18 cM (3/17, SOM Table 3). The sex locus has been mapped to a single location in other Hymenoptera such as *Bombus terrestris* (Gadau et al. 2001), *Bracon hebetor* (Antolin et al. 1996) and *Bracon* sp. near *hebetor* (Holloway et al. 2000). In the honey bee *csd* has not only been mapped (Hunt and Page 1995) and physically located on chromosome VIII (Beye and Moritz 1996), but also been isolated and characterized (Beye et al. 2003; Hasselmann et al. 2008; Gempe et al. 2009). The *vlp*-*p40* locus, that plays a role in protecting the wasp egg from its host immunity response (Hellers et al. 1996), is linked to marker *Vcan109* with a recombination distance of 4.7 cM (SOM Table 4). As *V. canescens* has *N* = 11 chromosomes to find one linked marker out of 27 tested is according to expectation. Hence, our results are consistent with a single and unlinked position of these two genes in the *V. canescens* genome.

We can compare the degree of homozygosity at the two loci *vlp*-*p40* and *csd* and their linked markers between arrhenotokous and thelytokous individuals. Complementary sex determination means that femaleness depends on heterozygosity of the *csd* locus. The *csd* locus is expected to be heterozygous in both arrhenotokous and thelytokous females. CSD is only compatible with thelytoky (all female production) if the *csd* locus is located in a region were heterozygosity is maintained, such as near a centromere or in an inversion (Beukeboom and Pijnacker 2000). The linked *Vcan071* marker is therefore expected to have a higher degree of heterozygosity in thelytokous individuals than unlinked markers. In this study, *Vcan071* as well as all other 14 microsatellites were homozygous in all tested thelytokous individuals. This could mean that the *csd* locus is highly recombining itself (Beye et al. 2003), or that linkage is not tight enough and sample sizes too small to detect heterozygosity. Malmberg et al. (2000) found among a sample of 102 arrhenotokous and 19 thelytokous females, 35 and zero heterozygotes respectively for *vlp*-*p40* (χ2 = 18.97, *df* = 2, *p* = 7.6E-05). A reduced heterozygosity of this gene in thelytokous individuals is in concordance with the *vlp*-*p40* locus undergoing genome homozygosation and it may therefore be located in a region of frequent recombination, such as distal from a centromere. In accordance, the linked marker *Vcan109* was found to be homozygous in all seven tested thelytokous females.

We have shown that the developed microsatellite markers for *V. canescens* are a useful tool for study of the genetic variability of arrhenotokous and thelytokous individuals. The markers can also be used for more detailed genetic studies in *V. canescens* in the future, for example Quantitative Trait Locus mapping of life history and behavioural traits. Arrhenotokous and thelytokous females have different foraging behaviour and oviposition strategies (e.g. Amat et al. 2006) and they allocate energy in different ways (e.g. Pelosse et al. 2010; Lukas et al. 2010). Genetic study of these traits would be informative for other species and increase our understanding of the genetics of adaptation in parasitoids.

## Electronic supplementary material

Below is the link to the electronic supplementary material.

Collection sites in South-Eastern France. Location abbreviations are indicated in SOM Table 1. Modified after Beukeboom et al. (1999) (PS 1047 kb)

Supplementary material 2 (DOCX 79 kb)
