# Supplementary material for: Genetic variability of arrhenotokous and thelytokous Venturiacanescens (Hymenoptera)
Source: Genetica. 2012 May 22;140(1):53–63. doi: 10.1007/s10709-012-9657-6 (PMC3386485; doi:10.1007/s10709-012-9657-6)
Supplement: Supplementary file 2 — Supplementary material 2 (DOCX 79 kb) [file 10709_2012_9657_MOESM2_ESM.docx]

**SOM Table 1**. Summary of samples used in this study. Indicated is the transect location number from SOM Figure 1, location name, collection year, reproductive mode, sample name and in which analysis the sample was used. A = arrhenotokous, T = thelytokous.

| ***Transect location*** | ***Location*** | ***Collection Year*** | ***Mode*** | ***Sample*** | ***Microsatellite***  ***data*** | ***Mitochondrial data*** |
| --- | --- | --- | --- | --- | --- | --- |
| Côte d’Azur (France) |  |  |  |  |  |  |
| 2 | Belvre l'Èze | 1997 | A | Bl'E A 97 | + | + |
| 4 | Cagnes sur Mer | 1997 | A | CsM A 97 | + | + |
| 3 | Cap d'Antibes INRA | 1997 | A | Cd'AI A 97 | + | + |
| 6 | Eze | 1997 | A | Eze A 98 | + | + |
| 5 | Gothéron INRA | 1997 | A | Got A 97 | + | + |
| 12 | Mont Boron | 1997 | A | MtB A 97 | + | + |
| 14 | Panoramèr | 1997 | A | Pmer A 97 | + | + |
|  |  | 1997 | A | PdC A 97 | + | + |
| 15 | Saint Jan | 1997 | A | StJ A 97 | + | + |
| 13 | Saint Laurent | 1997 | A | StL A 97 | + | + |
|  |  | 1997 | A | SV A 97 | + | + |
| 18 | Vallauris | 1997 | A | Vala A 98 | + | + |
| 26 | Villefranche | 1997 | A | VF A 98 | + | + |
| 1 | Anthéor | 1998 | A | Anth A 98 | + | + |
| 7 | La Brague | 1998 | A | LB A 98 | + | + |
| 23 | La Vallée Verte | 1998 | A | VV A 98 | - | + |
| 27 | Mont Gros | 1998 | A | MtG A 98 | + | - |
| 28 | Mont Vinagrier | 1998 | A | MtV A 98 | + | - |
| 14 | Panoramèr | 1998 | A | Pm A 98 | + | + |
| 19 | Saint Guitte | 1998 | A | StG A 98 | + | + |
| 3 | Antibes Botanique | 2003 | A | Ant Bot A 03 | + | + |
| 12 | Mont Boron | 2003 | A | MtB A 03 | + | + |
| 1 | Anthéor | 1997 | T | Anth T 97 | + | + |
| 14 | Panoramèr | 1997 | T | Pmer T 97 | + | + |
| 18 | Vallauris | 1997 | T | Val T 97 | + | + |
| 3 | Antibes Botanique | 2003 | T | Ant Bot T 03 | + | + |
| 12 | Mont Boron | 2003 | T | MtB T 03 | + | + |
| 24 | Valbonne | 2003 | T | Valb T 03 | + | + |
| Costa Blanca (Spain) |  |  |  |  |  |  |
|  | San Juan de Alicante | 2003 | T | SJ T 03 | + | + |

**SOM Table 2**. Overview of microsatellite markers of *Venturia canescens.* Size: expected size of the sequenced allele (bp); Repeat motif: repeat comprised between the primers; Tm: annealing temperature (ºC); Primer sequences: forward and reverse 5’-3’ primer sequence. Alleles: allele sizes observed; *N_A_:* the number of alleles per locus. na: not amplified

| ***Locus*** | ***Accession No.*** | ***Size (bp)*** | ***Repeat Motif*** | ***Tm (ºC)*** |  | ***Primer sequence***  ***(5’ – 3’)*** | ***Arrhenotokous***  ***Alleles*** | ***N_A_*** | ***Thelytokous***  ***Alleles*** | ***N_A_*** |
| --- | --- | --- | --- | --- | --- | --- | --- | --- | --- | --- |
|  |  |  |  |  |  |  |  |  |  |  |
| **Vcan060** | GU053668 | 244 | (GA)9 | 58 | F: | GGAGTTACTGAAGGCAAACAAGG | 253 247 | 2 | 247 249 | 2 |
|  |  |  |  |  | R: | AATGACTGAACGGGACTCGAT |  |  |  |  |
| **Vcan061** | GU053669 | 186 | (CT)13 | 55 | F: | TACGGTACTGAGAGATGTGTGGA | 178 184 188 190 192 194 200 | 7 | 188 190 | 2 |
|  |  |  |  |  | R: | TCGGTTAGAAGTGAGACCTGAAC |  |  |  |  |
| **Vcan062** | GU053670 | 254 | (CT)8 | 55 | F: | TGACTCCCTATGCACTTCTTCTC | 250 254 260 266 | 4 | 250 256 | 2 |
|  |  |  |  |  | R: | GAAGGTGTCGAGATATGGTGAAC |  |  |  |  |
| **Vcan063** | GU053671 | 179 | (AG)39 | 55 | F: | CGTATACTCACGCACACACAAAG | 174 178 182 184 186 | 5 | 168 182 | 2 |
|  |  |  |  |  | R: | CTTGACGATATGGGTTGATGG |  |  |  |  |
| **Vcan064** | GU053672 | 288 | Complex* | 55 | F: | GTTGCTAACTTCGAGGACAGACT | 277 280 283 286 289 295 | 6 | 289 | 1 |
|  |  |  |  |  | R: | AGTAGTAACGTTCGATGGCAGAG |  |  |  |  |
| **Vcan065** | GU053673 | 218 | (TGC)15 | 55 | F: | TCATTGTCACTGGTCGGTGT | 200 209 212 215 218 221 224 227 230 233 236 | 11 | 215 221 227 | 3 |
|  |  |  |  |  | R: | GATCATAGGCAACAGCAGCA |  |  |  |  |
| **Vcan066** | GU053674 | 245 | (CTTT)11 | 55 | F: | GATACCAGACTCGAGATCTATTCAA | 238 240 242 244 248 250 252 260 | 8 | 244 248 | 2 |
|  |  |  |  |  | R: | CGACCGACAATCAAGGTTTT |  |  |  |  |
| **Vcan067** | GU053675 | 155 | (CAA)26 | 55 | F: | ATGGTTCAGCAGCAACATCA | 139 142 151 154 157 160 | 6 | 148 151 154 157 | 4 |
|  |  |  |  |  | R: | GTTCCCTTGTAAAGCGGATG |  |  |  |  |
| **Vcan068** | GU053676 | 155 | (TTCG)21 | 58 | F: | TCCCGACTTTCTCACTCCTC | 141 161 165 169 177 | 5 | na | Na |
|  |  |  |  |  | R: | AGGAAGGAAGGAACGAAGGA |  |  |  |  |
| **Vcan069** | GU053677 | 228 | (AAC)45 | 55 | F: | GAATGAGGATCAGCAAAATCG | 199 218 220 223 226 229 | 6 | 229 | 1 |
|  |  |  |  |  | R: | GATGGCAGAGCAACTCGTTT |  |  |  |  |
| **Vcan070** | GU053678 | 228 | (GCT)10 | 55 | F: | TGCTCGCCCTTTTCTTTATT | 212 215 221 224 227 | 5 | 227 230 | 2 |
|  |  |  |  |  | R: | CATCTGCCACGACTCTCAAG |  |  |  |  |
| **Vcan071** | GU053679 | 232 | (CAA)11 | 55 | F: | CTCCTACGCACTCCCTTCAC | 228 231 234 237 240 243 246 252 | 8 | 222 225 231 243 | 4 |
|  |  |  |  |  | R: | TTGTACGTTGGCACTTGAGC |  |  |  |  |

* (CAA) CAG (CAA)8 CAG (CAA) CAG (CAA)4 CAG CAT (CAA)4 CCA (CAA)2 (CAG)4 (CAA) CAG (CAA)2 CAC (CAG)2 CAA CAG (CAA)3 CCA

**SOM Table 2**. Continued

| ***Locus*** | ***Accession No.*** | ***Size (bp)*** | ***Repeat Motif*** | ***Tm (ºC)*** |  | ***Primer sequence***  ***(5’ – 3’)*** | ***Arrhenotokous***  ***Alleles*** | ***Na*** | ***Thelytokous***  ***Alleles*** | ***Na*** |
| --- | --- | --- | --- | --- | --- | --- | --- | --- | --- | --- |
| **Vcan072** | GU053680 | 196 | (GAC)14 | 55 | F: | TGAATTTGTCGTTGCTGCTC | 200 203 | 2 | na | Na |
|  |  |  |  |  | R: | CGAGGAAGTTCAGGCTCAAG |  |  |  |  |
| **Vcan073** | GU053681 | 244 | (TGT)15 | 60 | F: | GGTCCAACGGTACTTCCTGA | 206 224 245 248 254 260 | 6 | 242 245 248 | 3 |
|  |  |  |  |  | R: | ACTTCCGTCAGCCCTACCTT |  |  |  |  |
| **Vcan074** | GU053682 | 198 | (AGA)8 | 61 | F: | CCGAAGCTGAAGAAATCGAA | 200 | 1 | Na | na |
|  |  |  |  |  | R: | CTACAGAGAGGGGCAAATCG |  |  |  |  |
| **Vcan075** | GU053683 | 193 | (CAA)10 | 60 | F: | TCGTCGGATCAACACAATTT | 193 | 1 | 190 193 | 2 |
|  |  |  |  |  | R: | TCAAGAATTCGGGAACATCC |  |  |  |  |
| **Vcan076** | GU053684 | 154 | (TGA)16 | 60 | F: | TTGACGCCGTCGAACTAATA | 156 | 1 | na | Na |
|  |  |  |  |  | R: | GTGAAACTGGAGCCTTTGGA |  |  |  |  |
| **Vcan077** | GU053685 | 195 | (GAC)3 | 62 | F: | GGTGGATTGACGTGCCTTAT | 196 | 1 | 193 196 | 2 |
|  |  |  |  |  | R: | CCCTCCCCACTTCTCTCTTT |  |  |  |  |
| **Vcan078** | GU053686 | 170 | (ATT)5 | 61 | F: | AGGTGATGTTAGGCGGTTTG | 168 171 | 2 | 168 | 1 |
|  |  |  |  |  | R: | TTTTCGCGGGTTTTGTTTAC |  |  |  |  |
| **Vcan079** | GU053687 | 157 | (GA)15 | 61 | F: | AGGAACGCAAAATGAAATGG | 146 156 158 | 3 | 154 160 | 2 |
|  |  |  |  |  | R: | TCGTTCGAACTTTTCCCCTA |  |  |  |  |
| **Vcan080** | GU053688 | 181 | (AGG)6 | 63 | F: | TCCATCTTCGCTCTTTTTCC | 181 | 1 | 181 | 1 |
|  |  |  |  |  | R: | CTTTCTCTTCGCGCTCACTT |  |  |  |  |
| **Vcan081** | GU053689 | 249 | (TGC)6 | 60 | F: | GCTAGACTCCACGGCTACCA | 251 | 1 | Na | na |
|  |  |  |  |  | R: | CAATAAGGGGCAATTCGTGA |  |  |  |  |
| **Vcan082** | GU053690 | 169 | (CAA)8 | 63 | F: | GCAACTCCTGCAACAACAGA | 169 | 1 | 169 172 | 1 |
|  |  |  |  |  | R: | GTGATGAGGGTAGCCGATGT |  |  |  |  |
| **Vcan083** | GU053691 | 250 | (TGA)11 | 60 | F: | ATAGCTCATCGCTCCTCTGC | 241 244 247 | 3 | 241 247 250 | 3 |
|  |  |  |  |  | R: | CGCCCATCTTGTGCTTATGT |  |  |  |  |
| **Vcan084** | GU053692 | 250 | (AGA)7 | 60 | F: | ACTCGATTTGCGTGGAAAAA | 250 | 1 | na | Na |
|  |  |  |  |  | R: | TTTCGCTTGTTGCTGAGTTC |  |  |  |  |
| **Vcan085** | GU053693 | 199 | (TTC)8 | 53 | F: | AGGTTCAATGGCTTTGCTGT | 146 161 197 200 | 4 | 197 200 | 21 |
|  |  |  |  |  | R: | GCTTTCCGAGCTTTTCCTCT |  |  |  |  |
| **Vcan086** | GU053694 | 244 | (AC)24 | 60 | F: | AGCAAACGGGACTTGAATTG | 245 | 1 | na | na |
|  |  |  |  |  | R: | GGGACTCCAAACCCTCTTGT |  |  |  |  |

**SOM Table 2**. Continued

| ***Locus*** | ***Accession No.*** | ***Size (bp)*** | ***Repeat Motif*** | ***Tm (ºC)*** |  | ***Primer sequence***  ***(5’ – 3’)*** | ***Arrhenotokous***  ***Alleles*** | ***Na*** | ***Thelytokous***  ***Alleles*** | ***Na*** |
| --- | --- | --- | --- | --- | --- | --- | --- | --- | --- | --- |
| **Vcan087** | GU053695 | 165 | (GT)23 | 55 | F: | CTTCTAGCTCCGTCGGTGTC | 165 | 1 | 165 | 1 |
|  |  |  |  |  | R: | CGGTGGTTGTTGTCGAGTTA |  |  |  |  |
| **Vcan088** | GU053696 | 198 | (CA)44 | 60 | F: | AGTAACCGGTCAGCCTTTGG | 132 136 142 144 | 4 | 132 134 136 142 | 4 |
|  |  |  |  |  | R: | CACGTTCCAATTTCCACACA |  |  |  |  |
| **Vcan089** | GU053697 | 234 | (TC)33 | 50 | F: | GGCCATGTTTCCTACTTCCA | 190 | 1 | na | na |
|  |  |  |  |  | R: | GGCGGAAAGATTTTTCGATAG |  |  |  |  |
| **Vcan090** | GU053698 | 243 | (CT)37 | 55 | F: | ATGCGGATGCGTAAGGATAG | 251 | 1 | na | na |
|  |  |  |  |  | R: | GCTGCTTAACGTTTCGGTCT |  |  |  |  |
| **Vcan091** | GU053699 | 184 | (GA)32 | 60 | F: | GTAGGCACGTACCGAGGAAA | 158 170 174 186 | 4 | 154 158 | 2 |
|  |  |  |  |  | R: | TCCACGCTCGTGTGTGTACT |  |  |  |  |
| **Vcan092** | GU053700 | 210 | (TTCC)6 | 60 | F: | CGTTCGTTCTTTCGTTCGTT | 208 212 216 220 | 4 | 212 216 220 | 3 |
|  |  |  |  |  | R: | CGGCATTGTCCTTCTTGTTT |  |  |  |  |
| **Vcan093** | GU053701 | 201 | (TC)21 | 50 | F: | CTACCAGCACGAGAAGCTGA | 201 | 1 | na | na |
|  |  |  |  |  | R: | TTTCTCGGTGCTTCTCCACT |  |  |  |  |
| **Vcan094** | GU053702 | 152 | (CT)26 | 60 | F: | ACGATCGCTCAATCGAAGTT | 156 158 | 2 | 154 156 | 2 |
|  |  |  |  |  | R: | CTCCCATAAACTCGGAGCAA |  |  |  |  |
| **Vcan095** | GU053703 | 100 | (CA)24 | 50 | F: | GTAATCATTTTCGCTCCGTGA | 080 092 096 | 3 | na | na |
|  |  |  |  |  | R: | TCGTTTCTCTTTTCGTTCGAG |  |  |  |  |
| **Vcan096** | GU053704 | 175 | (AG)39 | 55 | F: | CTCACGCACACACAAAGTCC | 172 176 178 | 3 | 176 | 1 |
|  |  |  |  |  | R: | TGCTTGACGATATGGGTTGA |  |  |  |  |
| **Vcan097** | GU053705 | 140 | (GA)15 | 55 | F: | AATGGAGACAACGAGGCAAC | 140 142 144 | 3 | 140 142 152 | 3 |
|  |  |  |  |  | R: | ATCAGAGTCGACCCAGCAAC |  |  |  |  |
| **Vcan098** | GU053706 | 233 | (GT)15 | 50 | F: | CAATTCGGAAACACTGCAAA | 233 | 1 | na | na |
|  |  |  |  |  | R: | CCGCTCGATCTTTTCATTTC |  |  |  |  |
| **Vcan099** | GU053707 | 123 | (AG)12 | 55 | F: | TGGCCATAACAGGGAGAAAG | 126 128 130 138 | 4 | 126 | 1 |
|  |  |  |  |  | R: | GTCACTGGGGAAGAGTGGTG |  |  |  |  |
| **Vcan100** | GU053708 | 224 | (GA)12 | 60 | F: | ACGGTCAAGTTACCCAAGCA | 215 | 1 | na | na |
|  |  |  |  |  | R: | GACCAAGCTCCGGATAAACA |  |  |  |  |
| **Vcan101** | GU053709 | 200 | (CT)15 | 45 | F: | ATCTGGACTAAGCGCGAGAG | 190 | 1 | na | na |
|  |  |  |  |  | R: | CAACGAGAGGGAAAGAGACG |  |  |  |  |

**SOM Table 2**. Continued

| ***Locus*** | ***Accession No.*** | ***Size (bp)*** | ***Repeat Motif*** | ***Tm (ºC)*** |  | ***Primer sequence***  ***(5’ – 3’)*** | ***Arrhenotokous***  ***Alleles*** | ***Na*** | ***Thelytokous***  ***Alleles*** | ***Na*** |
| --- | --- | --- | --- | --- | --- | --- | --- | --- | --- | --- |
| **Vcan102** | GU053710 | 174 | (TC)14 | 60 | F: | TTCCAATTCACGAATCAACG | 161 165 169 175 | 4 | 161 169 172 175 177 | 5 |
|  |  |  |  |  | R: | CCTCTGAGTCACCGAAAAGC |  |  |  |  |
| **Vcan103** | GU053711 | 206 | (TC)10 | 48 | F: | CTCAAGCTATGCATCCAACG | 154 | 1 | na | na |
|  |  |  |  |  | R: | TCTCGGAGTCAATCCCACTC |  |  |  |  |
| **Vcan104** | GU053712 | 164 | (GA)14 | 55 | F: | CAAAAGGGAGGGAAAGGAAG | 164 | 1 | na | na |
|  |  |  |  |  | R: | CCCACGTTTTCGGTGTACTT |  |  |  |  |
| **Vcan105** | GU053713 | 188 | (TC)15 | 48 | F: | TGGGCAATTACCCCACTAAA | 156 | 1 | na | na |
|  |  |  |  |  | R: | GCACGTGCAATTCTGATGAC |  |  |  |  |
| **Vcan106** | GU053714 | 197 | (TC)24 | 60 | F: | CCTCATCTCGAGGGAGGATT | 190 196 206 208 210 214 224 | 7 | 186 188 190 194 210 | 5 |
|  |  |  |  |  | R: | ATCGCGAGTTGCGTAGTTTC |  |  |  |  |
| **Vcan107** | GU053715 | 282 | (AC)13 | 60 | F: | GTCGCCGGCTCATATTTTTA | 283 | 1 | na | na |
|  |  |  |  |  | R: | AGCAAGTCTGCGATCTTTCC |  |  |  |  |
| **Vcan108** | GU053716 | 188 | (TC)24 | 50 | F: | GGATACACGAACCTGGCATT | 190 | 1 | na | na |
|  |  |  |  |  | R: | AGACCGAGAGAGGAGGAAGG |  |  |  |  |
| **Vcan109** | GU053717 | 192 | (AG)10 | 60 | F: | TTAATTGAACGGGGAAAACG | 189 191 193 | 3 | 193 | 1 |
|  |  |  |  |  | R: | GCAGTCGGTGTAGCGTGTTA |  |  |  |  |
| **Vcan110** | GU053718 | 172 | (AC)25 | 55 | F: | CCATTCATTCGGATCTCACC | 172 174 | 2 | 172 | 1 |
|  |  |  |  |  | R: | CCGACGTTTGTATCTTCGTTC |  |  |  |  |
| **Vcan111** | GU053719 | 264 | (ACC)12 | 55 | F: | CCACACGAACAATGTCAAT | 264 | 1 | na | na |
|  |  |  |  |  | R: | CCGTTTTTATGAGCGTAGAG |  |  |  |  |
| **Vcan112** | GU053720 | 159 | (TC)15 | 55 | F: | GCAGAGATTTTTGCCACAGG | 143 147 149 151 161 172 176 178 | 8 | 143 149 161 | 3 |
|  |  |  |  |  | R: | TGGCTGGATGAAGGGATATT |  |  |  |  |
| **Vcan113** | GU053721 | 199 | (GA)11 | 55 | F: | TTCAGGGAGGATAGAACGTA | 199 | 1 | 199 | 1 |
|  |  |  |  |  | R: | TCTCTCTCCTTCCCTTTCTC |  |  |  |  |
| **Vcan114** | GU053722 | 241 | (AG)10 | 55 | F: | AAAAATGAACGACAGAAGGA | 237 241 245 247 251 | 5 | 243 | 1 |
|  |  |  |  |  | R: | GTTGCGCTCTTTGTGAATA |  |  |  |  |
| **Vcan115** | GU053723 | 106 | (CT)11 | 55 | F: | TTTTTCACTCTTCGTTCCTC | 096 104 106 124 | 4 | 104 110 | 2 |
|  |  |  |  |  | R: | TGCTACCCTCTTGATCTCC |  |  |  |  |

**SOM Table 3**. Recombination between the *csd* locus and microsatellite marker *Vcan071* from a cross between a heterozygous female and a hemizygous male with a matched allele for the marker. Diploid males are significantly more often homozygous for *Vcan071*.

| *genotype* | *Heterozygous*  *Vcan071* | *Homozygous*  *Vcan071* | *P* |
| --- | --- | --- | --- |
| 2n females | 6 | 8 |  |
| 2n males | 3 | 14 | 0.01<P<0.025 |

**SOM Table 4**. Linkage between the *vlp-p40* locus (alleles + and -) and microsatellite marker *Vcan109* (alleles m=maternal and p=paternal)

| *vlp*  *Vcan109* | *+* | *-* | *total* |
| --- | --- | --- | --- |
| m | 38 | 2 | 40 |
| p | 1 | 23 | 24 |
| **total** | 39 | 25 | 64 |

X^2^=48,246, df=1, p<0.0001, N=64

**SOM Table 5** Genotyping raw data for 15 polymorphic microsatellites of the 29 field collected strains used in this study.

| Indv | Location | | Year | Vcan061 | Vcan062 | Vcan063 | Vcan064 | Vcan065 | Vcan066 | Vcan067 | Vcan069 | Vcan070 | Vcan071 | Vcan097 | Vcan109 | Vcan110 | Vcan112 | Vcan114 |
| --- | --- | --- | --- | --- | --- | --- | --- | --- | --- | --- | --- | --- | --- | --- | --- | --- | --- | --- |
|  |  | **Arrhenotokous** | |  |  |  |  |  |  |  |  |  |  |  |  |  |  |  |
| 1 | Bl'E | | 1997 | 188190 | 254 | 182184 | 286 | 230233 | 240252 | 0 | 223226 | 224 | 0 | 142 | 0 | 0 | 143149 | 0 |
| 2 | StJ | | 1997 | 190192 | 254266 | 182 | 286 | 209218 | 248 | 142154 | 226 | 220223 | 0 | 140 | 189 | 0 | 143 | 241251 |
| 3 | Cd'Al | | 1997 | 188190 | 250 | 182184 | 280286 | 227236 | 240244 | 0 | 220226 | 224 | 228234 | 140 | 0 | 172 | 143149 | 247251 |
| 4 | StL | | 1997 | 188190 | 250266 | 182 | 286295 | 233 | 240244 | 157 | 0 | 224 | 228234 | 140 | 189191 | 172 | 143 | 247 |
| 5 | CsM | | 1997 | 184 | 250266 | 0 | 286 | 224233 | 244 | 157 | 226 | 215 | 0 | 140142 | 189191 | 172 | 143149 | 247 |
| 6 | SV | | 1997 | 190200 | 260266 | 174184 | 286 | 215230 | 252260 | 151 | 226 | 224 | 0 | 140142 | 189 | 0 | 149 | 241 |
| 7 | Got | | 1997 | 190200 | 260266 | 0 | 280286 | 212230 | 240244 | 151 | 0 | 224 | 231 | 142 | 191 | 172 | 149161 | 237241 |
| 8 | Mt.B | | 1997 | 188190 | 250260 | 182 | 283289 | 218221 | 240 | 154160 | 223229 | 0 | 234 | 140 | 191193 | 0 | 143149 | 241 |
| 9 | PdC | | 1997 | 188190 | 250254 | 182184 | 286289 | 209233 | 240 | 142 | 226229 | 0 | 234 | 140 | 189191 | 174 | 143 | 247 |
| 10 | Pmer | | 1997 | 188190 | 250254 | 182 | 286 | 212227 | 240244 | 0 | 226 | 220223 | 0 | 140144 | 189193 | 172 | 143149 | 241247 |
| 11 | Eze | | 1998 | 188200 | 250266 | 182184 | 277286 | 218 | 240244 | 142160 | 217226 | 223226 | 237 | 140142 | 191 | 172 | 143 | 247 |
| 12 | LB | | 1998 | 178 | 250266 | 182 | 283286 | 221227 | 240244 | 157 | 223226 | 224 | 0 | 140144 | 191 | 172 | 143 | 241247 |
| 13 | MtG | | 1998 | 188 | 266 | 182 | 286 | 209212 | 240244 | 157 | 226 | 0 | 237243 | 0 | 189191 | 172 | 143 | 241247 |
| 14 | MtV | | 1998 | 188190 | 250266 | 182 | 280286 | 209218 | 240252 | 139 | 220226 | 223 | 0 | 140142 | 191193 | 174 | 147161 | 247251 |
| 15 | Anth | | 1998 | 188 | 266 | 182186 | 286 | 230 | 244 | 142 | 226 | 212220 | 234 | 140142 | 189191 | 172 | 143 | 241 |
| 16 | Pm | | 1998 | 190 | 260266 | 182186 | 286 | 218230 | 240244 | 142154 | 226 | 212220 | 0 | 140 | 189191 | 172 | 143 | 241247 |
| 17 | StG | | 1998 | 188190 | 266 | 182 | 286 | 212227 | 240244 | 0 | 0 | 220223 | 234 | 140 | 191 | 172 | 143 | 241 |
| 18 | Vala | | 1998 | 190 | 250266 | 182186 | 283286 | 215224 | 240260 | 0 | 223226 | 222 | 246 | 140142 | 191 | 172 | 143 | 247 |
| 19 | VF | | 1998 | 190194 | 250 | 182186 | 286 | 209227 | 240244 | 154157 | 226 | 220223 | 246 | 140 | 189193 | 172 | 143 | 0 |
| 20 | VV | | 1998 | 0 | 0 | 0 | 0 | 0 | 0 | 0 | 0 | 222 | 0 | 0 | 0 | 0 | 0 | 0 |
| 21 | Ant Bot | | 2003 | 188 | 250260 | 182184 | 286 | 212227 | 240 | 139154 | 226 | 222 | 240 | 140 | 191193 | 172 | 143 | 241 |
| 22 | MtB | | 2003 | 188190 | 266 | 182 | 283286 | 200227 | 244 | 154160 | 223226 | 224 | 0 | 140 | 189191 | 172 | 143 | 241247 |
|  |  | **Thelytokous** | |  |  |  |  |  |  |  |  |  |  |  |  |  |  |  |
| 23 | Anth | | 1997 | 188 | 256 | 182 | 289 | 221 | 248 | 157 | 229 | 0 | 231 | 142 | 193 | 172 | 161 | 243 |
| 24 | Pmer | | 1997 | 188 | 256 | 182 | 289 | 221 | 248 | 157 | 229 | 227 | 231 | 142 | 193 | 0 | 161 | 243 |
| 25 | Val | | 1997 | 188 | 256 | 182 | 286 | 221 | 248 | 157 | 229 | 230 | 0 | 142 | 193 | 172 | 0 | 243 |
| 26 | Ant Bot | | 2003 | 188 | 256 | 182 | 289 | 221 | 248 | 157 | 229 | 230 | 231 | 142 | 193 | 172 | 161 | 243 |
| 27 | Valb | | 2003 | 188 | 256 | 182 | 289 | 221 | 248 | 157 | 0 | 230 | 231 | 142 | 193 | 172 | 161 | 243 |
| 28 | MtB | | 2003 | 188 | 256 | 182 | 289 | 221 | 248 | 157 | 229 | 230 | 231 | 142 | 193 | 172 | 161 | 243 |
| 29 | SJ | | 2003 | 190 | 250 | 182 | 0 | 215 | 244 | 151 | 229 | 227 | 243 | 152 | 193 | 172 | 161 | 0 |

**SOM Table 6**. Observed allele frequencies in arrhenotokous and thelytokous females for 15 microsatellites. N designates de number of individuals tested of each reproductive mode.

| Locus | Alleles | Arrhenotokous | Thelytokous |  | Locus | Alleles | Arrhenotokous | Thelytokous |
| --- | --- | --- | --- | --- | --- | --- | --- | --- |
| *Vcan*61 | N | 21 | 7 |  | *Vcan*69 |  | 18 | 6 |
|  | 178 | 0.048 | 0 |  |  | 217 | 0.028 | 0 |
|  | 184 | 0.048 | 0 |  |  | 220 | 0.056 | 0 |
|  | 188 | 0.381 | 0.857 |  |  | 223 | 0.139 | 0 |
|  | 190 | 0.405 | 0.143 |  |  | 226 | 0.722 | 0 |
|  | 192 | 0.024 | 0 |  |  | 229 | 0.056 | 1 |
|  | 194 | 0.024 | 0 |  | *Vcan*70 |  | 19 | 6 |
|  | 200 | 0.071 | 0 |  |  | 212 | 0.053 | 0 |
| *Vcan*62 |  | 21 | 7 |  |  | 215 | 0.053 | 0 |
|  | 250 | 0.333 | 0.143 |  |  | 221 | 0.316 | 0 |
|  | 254 | 0.119 | 0 |  |  | 224 | 0.553 | 0 |
|  | 256 | 0 | 0.857 |  |  | 227 | 0.026 | 0.333 |
|  | 260 | 0.119 | 0 |  |  | 230 | 0 | 0.667 |
|  | 266 | 0.429 | 0 |  | *Vcan*71 |  | 12 | 6 |
| *Vcan*63 |  | 19 | 7 |  |  | 228 | 0.083 | 0 |
|  | 174 | 0.026 | 0 |  |  | 231 | 0.083 | 0.833 |
|  | 182 | 0.711 | 1 |  |  | 234 | 0.417 | 0 |
|  | 184 | 0.158 | 0 |  |  | 237 | 0.125 | 0 |
|  | 186 | 0.105 | 0 |  |  | 240 | 0.083 | 0 |
| *Vcan*64 |  | 21 | 6 |  |  | 243 | 0.042 | 0.167 |
|  | 277 | 0.024 | 0 |  |  | 246 | 0.167 | 0 |
|  | 280 | 0.071 | 0 |  | *Vcan*97 |  | 20 | 7 |
|  | 283 | 0.095 | 0 |  |  | 140 | 0.700 | 0 |
|  | 286 | 0.738 | 0.167 |  |  | 142 | 0.250 | 0.857 |
|  | 289 | 0.048 | 0.833 |  |  | 144 | 0.050 | 0 |
|  | 295 | 0.024 | 0 |  |  | 152 | 0 | 0.143 |
| *Vcan*65 |  | 21 | 7 |  | *Vcan*109 |  | 19 | 7 |
|  | 200 | 0.024 | 0 |  |  | 189 | 0.342 | 0 |
|  | 209 | 0.119 | 0 |  |  | 191 | 0.526 | 0 |
|  | 212 | 0.119 | 0 |  |  | 193 | 0.132 | 1 |
|  | 215 | 0.048 | 0.143 |  | *Vcan*110 |  | 17 | 6 |
|  | 218 | 0.143 | 0 |  |  | 172 | 0.882 | 1 |
|  | 221 | 0.048 | 0.857 |  |  | 174 | 0.118 | 0 |
|  | 224 | 0.048 | 0 |  | *Vcan*112 |  | 21 | 6 |
|  | 227 | 0.167 | 0 |  |  | 143 | 0.738 | 0 |
|  | 230 | 0.143 | 0 |  |  | 147 | 0.024 | 0 |
|  | 233 | 0.119 | 0 |  |  | 149 | 0.190 | 0 |
|  | 236 | 0.024 | 0 |  |  | 161 | 0.048 | 1 |
| *Vcan*66 |  | 21 | 7 |  | *Vcan*114 |  | 19 | 6 |
|  | 240 | 0.452 | 0 |  |  | 237 | 0.026 | 0 |
|  | 244 | 0.381 | 0.143 |  |  | 241 | 0.447 | 0 |
|  | 248 | 0.048 | 0.857 |  |  | 243 | 0 | 1 |
|  | 252 | 0.071 | 0 |  |  | 247 | 0.447 | 0 |
|  | 260 | 0.048 | 0 |  |  | 251 | 0.079 | 0 |
| *Vcan*67 |  | 16 | 7 |  |  |  |  |  |
|  | 139 | 0.094 | 0 |  |  |  |  |  |
|  | 142 | 0.219 | 0 |  |  |  |  |  |
|  | 151 | 0.125 | 0.143 |  |  |  |  |  |
|  | 154 | 0.188 | 0 |  |  |  |  |  |
|  | 157 | 0.281 | 0.857 |  |  |  |  |  |
|  | 160 | 0.094 | 0 |  |  |  |  |  |
